# *Trypanosoma* (*Herpetosoma*) diversity in rodents and lagomorphs of New Mexico with a focus on epizootological aspects of infection in Southern Plains woodrats (*Neotoma micropus*)

**DOI:** 10.1371/journal.pone.0244803

**Published:** 2020-12-31

**Authors:** Irina Goodrich, Clifton McKee, Michael Kosoy

**Affiliations:** 1 Centers for Disease Control and Prevention, Fort Collins, Colorado, United States of America; 2 Graduate Degree Program in Ecology, Colorado State University, Fort Collins, Colorado, United States of America; 3 Department of Biology, Colorado State University, Fort Collins, Colorado, United States of America; Universidade Federal de Minas Gerais, BRAZIL

## Abstract

Protozoan parasites of the genus *Trypanosoma* infect a broad diversity of vertebrates and several species cause significant illness in humans. However, understanding of the phylogenetic diversity, host associations, and infection dynamics of *Trypanosoma* species in naturally infected animals is incomplete. This study investigated the presence of *Trypanosoma* spp. in wild rodents and lagomorphs in northern New Mexico, United States, as well as phylogenetic relationships among these parasites. A total of 458 samples from 13 rodent and one lagomorph species collected between November 2002 and July 2004 were tested by nested PCR targeting the 18S ribosomal RNA gene (18S rRNA). *Trypanosoma* DNA was detected in 25.1% of all samples, with the highest rates of 50% in *Sylvilagus audubonii*, 33.1% in *Neotoma micropus*, and 32% in *Peromyscus leucopus*. Phylogenetic analysis of *Trypanosoma* sequences revealed five haplotypes within the subgenus *Herpetosoma* (*T*. *lewisi* clade). Focused analysis on the large number of samples from *N*. *micropus* showed that *Trypanosoma* infection varied by age class and that the same *Trypanosoma* haplotype could be detected in recaptured individuals over multiple months. This is the first report of *Trypanosoma* infections in *Dipodomys ordii* and *Otospermophilus variegatus*, and the first detection of a haplotype phylogenetically related to *T*. *nabiasi* in North America in *S*. *audubonii*. This study lends important new insight into the diversity of *Trypanosoma* species, their geographic ranges and host associations, and the dynamics of infection in natural populations.

## Introduction

*Trypanosoma* (class Kinetoplastida) is a diverse genus of flagellate blood parasites found in all groups of vertebrates [1]. Approximately 500 *Trypanosoma* species in dozens of lineages have been characterized and named based on morphological features, host associations, and DNA sequencing [2–7]. Trypanosomes are transmitted between vertebrate hosts by blood-feeding ectoparasites and micropredators. Trypanosomes of mammals fall into two biological groups based on the development of parasites in the vector and the mode of transmission from vector to host. The salivarian trypanosomes, including the causative agent of African sleeping sickness in humans, *T*. *brucei*, develop in the midgut of an insect vector, migrate to the salivary glands or proboscis, and pass to the host in the saliva. The stercorarian trypanosomes, including the agent of Chagas disease in the Americas, *T*. *cruzi*, develop in the hind gut of the vector and are transmitted through ingestion of the vector by the host or contamination of bite wounds with vector feces [1, 8]. Trypanosomes are also grouped based on their pathogenicity in their hosts, with some species causing significant disease in humans and domestic animals (e.g., *T*. *brucei*, *T*. *congolense*, *T*. *brucei*) and others appearing to cause no disease in their hosts [9, 10]. Finally, trypanosomes are classified into subgenera and other clades based on phylogenetic analysis of molecular sequences [2–7].

The stercorarian trypanosomes in the subgenus *Herpetosoma* (*T*. *lewisi* clade) are found in numerous rodent and lagomorph species globally [11]. At least 30 species from the clade have been named based on assumed host-specificity, but many species names require validation with DNA sequencing because they are morphologically similar [12–14]. While this group is often considered to be non-pathogenic [1], *T*. *lewisi* and *T*. *lewisi*-like parasites have been linked to illness in some rodent hosts [9, 15] or in humans that become infected through contact with hosts or vectors [16–18]. Given the zoonotic potential of these parasites, there is a need to characterize the diversity of *Trypanosoma* species in vertebrate hosts and understand the ecology of these parasites within natural systems where humans could be exposed [19].

Within the United States, there is an added concern about the circulation of *T*. *cruzi* in natural hosts and the potential for zoonotic transmission. Cases of Chagas disease linked to autochthonous vector-borne transmission have been reported in humans in several western and southern states (California, Arizona, Texas, Louisiana, Mississippi, Tennessee), but there is limited information on the circulation of this species or other trypanosomes in potential reservoir hosts, especially rodents and lagomorphs [20, 21]. *T*. *cruzi* has been reported in *Neotoma* spp. woodrats in California, Arizona, New Mexico, and Louisiana [22–28], but it is unclear whether the methods used in these studies could have detected other *Trypanosoma* species. *T*. *lewisi*-like trypanosomes *T*. *neotomae* and *T*. *kansasensis* have also been observed in blood from woodrats in California and Kansas [29, 30], but these parasites were only characterized morphologically and sequence information is therefore lacking. Surveys in Alaska have detected DNA sequences similar to *T*. *microti* in voles and lemmings in Alaska [31]. Finally, *T*. *nabiasi* infects European rabbits (*Oryctolagus cuniculus*) in Portugal, Spain, France, the UK, and Italy, and was introduced to Australia through the importation of *O*. *cuniculus* or its fleas [1, 32–34]. However, surveys of trypanosomes in rabbits in the US using molecular methods are yet to be published. Therefore, more surveys of trypanosomes in wild rodent and lagomorphs are necessary for surveillance of *T*. *cruzi* and to understand the prevalence and phylogenetic diversity of *Trypanosoma* parasites in this region.

As part of a surveillance study on vector-borne pathogens in New Mexico, the objectives of the study were to use molecular methods to estimate the prevalence of *Trypanosoma* infection in rodents and lagomorphs and evaluate the genetic diversity and host associations of detected *Trypanosoma* haplotypes. We hypothesized that multiple rodent species would be infected with *T*. *cruzi* or other trypanosomes within the *T*. *lewisi* clade and that haplotypes would exhibit a host-specific pattern, infecting either a single host or multiple related hosts. Previous studies on *Bartonella* infection in this same community showed high prevalence of infection in multiple species [35, 36], so we hypothesized that immune suppression associated with *Bartonella* infection might lead to a higher likelihood of *Bartonella*-infected animals carrying trypanosomes. Therefore, we analyzed coinfection rates across species. Finally, due to the large number of samples collected from one species, *Neotoma micropus*, we examined whether characteristics such as age, sex, flea burden, or *Bartonella* infection could explain variation in the probability of *Trypanosoma* infection across individuals. This study reveals previously unknown diversity of *Trypanosoma* parasites in New Mexico and provides new information on the infection dynamics of trypanosomes in *N*. *micropus*.

## Material and methods

### Sample collection and DNA extraction

Animals were captured in the Eldorado subdivision of Santa Fe County, New Mexico, United States (latitude: 35.30373 to 35.31617, longitude: -105.5681 to -105.57782), from November 2002 to July 2004 as part of a mark-recapture plague surveillance study [35]. The trapping sites were in pinyon-juniper woodlands in a suburban area nearby to homes and a recreational hiking area. For the purpose of broad surveillance of pathogens in rodents and lagomorphs, convenience sampling was implemented using a variety of traps: (1) small Sherman traps (2”x2”x6.5”) for mouse-sized rodents (HB Sherman Trap Company, Tallahassee, FL), (2) large Sherman traps (3”x3.5”x9”) for rat-sized rodents, and (3) Tomahawk traps (4”x4”x10”) for squirrel-sized rodents and rabbits (Tomahawk Live Trap Company, Hazelhurst, WI). Trapping locations were chosen based on prior identification of woodrat nests. Traps were baited with oats and peanut butter in the afternoon and checked the following morning, and any captured animals were then processed at a centralized location.

Animals were anaesthetized with isoflurane and a small sample of blood was collected (100–200 μl) by retro-orbital bleed with heparinized microhematocrit capillary tubes (MWI Veterinary Supply Company, Denver, CO) and stored in EDTA-treated tubes. Animal species were distinguished morphologically using available field guides [37] and keys developed by the University of New Mexico Museum of Southwestern Biology for the study area. Additionally, data on sex, weight, and age class were recorded for each animal. Captured animals were marked with ear tags and/or subcutaneous transponders (AVID, Folsom, LA) and then released at the location of capture. All animal handling procedures were approved by the Centers for Disease and Control and Prevention Division of Vector-Borne Diseases (CDC DVBD) Institutional Animal Care and Use Committee, protocol number 06–008. Blood samples were placed on dry ice in the field and then kept at -80°C in the laboratory until processing. Genomic DNA was extracted from 100 μl of blood using the KingFisher Flex Purification System and the associated MagMax Pathogen RNA/DNA Kit (ThermoFisher, Waltham, MA) according to the manufacturer’s protocol. The DNA was eluted in 90 μl of elution buffer and stored at 4°C while samples were being tested. The remaining blood samples have been archived at the CDC DVBD in Fort Collins, CO.

### *Trypanosoma* spp. detection, sequencing, and phylogenetic analysis

Two PCR protocols were used to test for the presence of *Trypanosoma* DNA in samples. We first tested samples for *T*. *cruzi* DNA with species-specific qPCR targeting the kinetoplast minicircle [38]. The protocol for qPCR used the forward primer Cruzi 1: ASTCGGCTGATCGTTTTCGA, the reverse primer Cruzi 2: AATTCCTCCAAGCAGCGGATA, and the probe Cruzi 3: CACACACTGGACACCAA. Positive (*T*. *cruzi*) and negative (deionized water) controls were included in all reactions to ensure that PCR worked properly. A second test to detect *Trypanosoma* spp. DNA targeting a fragment (about 530 bp) of the small subunit ribosomal RNA gene (18S rRNA) used external primers TRY927F: CAGAAACGAAACACGGGAG and TRY927R: CCTACTGGGCAGCTTGGA and internal primers SSU561F: TGGGATAACAAAGGAGCA and SSU561R: CTGAGACTGTAACCTCAAAGC [39]. The 18S rRNA gene was chosen because previous analyses have demonstrated that this marker is adequate for phylogenetic differentiation of *Trypanosoma* species [40]. Multiple studies have used these primers to amplify *Trypanosoma* DNA from clades across the *Trypanosoma* phylogeny associated with mammals, sauropsids, amphibians, and fish [39, 41–44]. A study by Hodo et al. [45] was also able to amplify DNA from the related trypanosomatid genus *Blastocrithidia* in the blood of bats with these 18S rRNA primers. Therefore, we assumed that the nested primers were sufficiently sensitive to amplify a broad diversity of trypanosomes that might be present in our samples. *T*. *lewisi* DNA was used for the positive control for the 18S rRNA nested PCR and deionized water was the negative control. No internal control targeting host DNA was used to identify false negatives in PCR tests. Extractions and PCR were performed in separate laboratories to minimize contamination.

Amplified fragments of the 18S rRNA gene were separated in 1.5% agarose gels stained with Biotium GelGreen (Biotium, Hayward, CA). Positive amplicons were purified using the QIAquick PCR Purification Kit (QIAGEN, Valencia, CA) according to the manufacturer’s protocol and sequenced in both directions with the internal primers (561F, 561R) on an Applied Biosystems Model 3130 Genetic Analyzer (Applied Biosystems, Foster City, CA). We assembled forward and reverse sequences using the SeqMan Pro program in Lasergene v12 (DNASTAR, Madison, WI). BLAST search was used to check whether the obtained sequences belonged to trypanosomes and to collect similar sequences from the GenBank database for the phylogenetic analysis. Amplified sequences from samples and reference sequences were aligned with MAFFT v7 [46] using the iterative local alignment method with generalized affine gap costs (E-INS-i) with default settings (https://mafft.cbrc.jp/alignment/server/). The alignment was manually inspected and adjusted, trimming the ends of sequences to match the newly obtained 18S rRNA sequences.

Novel *Trypanosoma* haplotypes were identified based on comparison with other sequences from this study and existing reference *Trypanosoma* sequences via maximum likelihood (ML) phylogenetic analysis using MEGA v7.0.26 [47]. An initial ML tree was estimated using the GTR+I+G model with four gamma categories. Identical sequences were labeled as distinct haplotypes and the sequence alignment was reduced to a single sequence per haplotype for final phylogenetic analysis. Phylogenetic model selection and phylogenetic reconstruction were performed with IQ-Tree v2.1.1 using the final alignment [48, 49]. The top-ranking model based on the Akaike information criterion with a correction for finite sample sizes (AICc) was chosen for ML phylogenetic analysis [50, 51]. The top model for the 18S rRNA alignment was a transition model with unequal base frequencies and two substitution rate categories (TIM2+F+R2). Branch support across the phylogeny was estimated with 1000 ultrafast bootstrap replicates [52]. The consensus tree was viewed and annotated using the *ape*, *phytools*, and *ggtree* packages in R v4.0.3 [53–56].

### *Trypanosoma*-*Bartonella* coinfection

To assess the occurrence of coinfection by *Trypanosoma* parasites and *Bartonella* bacteria in captured animals, we included information on the presence of *Bartonella* DNA in collected samples from our previous study [36]. In brief, *Bartonella* DNA was detected using a genus-specific qPCR targeting the *Bartonella* transfer-messenger RNA gene (*ssrA*) [57] and conventional PCR targeting the 16S-23S rRNA gene intergenic transcribed spacer (ITS) [58]. Samples that were positive by *ssrA* or ITS were tested by nested PCR for the citrate synthase gene (*gltA*) [59, 60], followed by sequencing and phylogenetic analysis of amplicons. Samples were considered *Bartonella* positive if they tested positive for at least two out of three targets (*ssrA*, ITA, and *gltA*) and were successfully sequenced.

### Statistical analysis

The prevalence of *Trypanosoma* and *Bartonella* DNA in tested samples was estimated for each species across all months using the number of infected animals out of the total sampled between November 2002 and July 2004. Our convenience sampling resulted in numerous recaptures of individual animals over multiple months. We did not remove or attempt to randomize the selection of samples collected from recaptured animals when estimating *Trypanosoma* or *Bartonella* DNA prevalence by host species. Confidence intervals (95% CI) for *Trypanosoma* prevalence in host species over each month when a species was captured were calculated with Wilson score intervals [61]. Host specificity of *Trypanosoma* haplotypes identified in positive animals was examined by plotting the relative abundance of each haplotype (i.e., the percent of all infections attributed to a haplotype) by host species. Differences in the *Trypanosoma* prevalence between species were analyzed using a chi-square test of proportions in R [55]. For all statistical tests, α < 0.05 was used as the cutoff for statistical significance.

We investigated the effects of temporal and ecological predictors on *Trypanosoma* prevalence in *N*. *micropus* because it was captured in all months and resulted in a large sample size for this species; other species had insufficient samples for such analysis. We performed model selection to choose the best fitting set of covariates that explained variation in prevalence. Within this model selection procedure, we also performed stratified sampling by individual ID number (without replacement) to choose only one sample from recaptured animals. The joint procedure involved stratified sampling of *N*. *micropus* individuals followed by model selection based on AICc [51] using the R package *MuMIn* [62] and was repeated 1000 times to account for individual random effects. Model selection was performed on fitted generalized linear models (GLM) using covariates of infection at the time of capture including date of capture, sex, age class (adult, subadult, or juvenile), body weight, the presence/absence of fleas, flea count, and *Bartonella* infection status. We included these predictors based on previous studies of rodent-borne trypanosomes that found relationships between *Trypanosoma* infection and age, weight, and flea burden [19, 63–67]. Our analysis of *Bartonella* infection in this same population of *N*. *micropus* found a significant interaction effect of sex and weight [36]. Therefore, we added this interaction as a potential covariate. Information on all individual covariates can be found in S1 Dataset. The mean AICc values were then calculated over all 1000 iterations of resampling for each model including different combinations of main effects and interaction effects. The model with the lowest mean AICc was chosen as the top model for explaining variation in prevalence.

Using the top GLM, we then estimated the probability of infection for *N*. *micropus* individuals based on the covariates included in the model. For this procedure, we again performed stratified sampling by individual tag number before fitting the GLM and estimated 95% CI for fixed effects in the model. The fit of the model was assessed using the coefficient of determination (R^2^) and the area under the receiver operating characteristic curve (AUC) using the R package *pROC* [68]. The process of stratified sampling and model fitting was repeated 1000 times and mean values were calculated for the probability of infection, upper and lower 95% CI, R^2^, and AUC.

The prevalence of coinfection with *Trypanosoma* and *Bartonella* was analyzed using a binomial test comparing the observed coinfection rate and an expected rate based on the product of the *Trypanosoma* and *Bartonella* infection rates. This test was performed on all species across all months and on separate species across all months. We also ran tests on those species with adequate sampling across dates (greater than one sample in a month and infection with *Trypanosoma*, *Bartonella*, or both).

## Results

### Sampled and recaptured animals

A total of 473 samples were collected from captured animals from November 2002 to July 2004 comprising 14 rodent and lagomorph species in 5 families (Table 1). Out of the total captured animals, 280 uniquely tagged individuals were identified, 67 (23.9%) of which were recaptured at least once over multiple months. After accounting for the recaptured individuals, the most abundant species among the captured individual animals were *Neotoma micropus* (46.8%), *Dipodomys ordii* (13.2%), and *Neotoma albigula* (10%). The recaptured animals represented five species: *N*. *micropus* (49), *N*. *albigula* (8), *D*. *ordii* (5), *Otospermophilus variegatus* (4), and *Peromyscus maniculatus* (1).

**Table 1:** Rodents and lagomorphs captured in the Eldorado subdivision of Santa Fe County, New Mexico, USA, from November 2002 to July 2004.

Family	Common name	Latin name	Samples	Tested samples	Tagged individuals	*Trypanosoma* DNA positive (%)	*Bartonella* DNA positive (%)	Coinfection positive (%)
Cricetidae	White-throated woodrat	*Neotoma albigula*	50	47	28	0 (0)	37 (78.7)	0 (0)
	Southern Plains woodrat	*Neotoma micropus*	272	263	131	87 (33.1)	196 (74.5)	68 (25.9)
	Northern grasshopper mouse	*Onychomys leucogaster*	8	8	7	0 (0)	7 (87.5)	0 (0)
	White-footed mouse	*Peromyscus leucopus*	25	25	23	8 (32)	12 (48)	3 (12)
	Deer mouse	*Peromyscus maniculatus*	14	14	9	1 (7.1)	3 (21.4)	0 (0)
	Pinyon mouse	*Peromyscus truei*	12	11	12	0 (0)	2 (18.2)	0 (0)
	Western harvest mouse	*Reithrodontomys megalotis*	2	2	1	0 (0)	1 (50)	0 (0)
Heteromyidae	Ord’s kangaroo rat	*Dipodomys ordii*	43	41	37	7 (17.1)	29 (70.7)	5 (12.2)
	Banner-tailed kangaroo rat	*Dipodomys spectabilis*	2	2	2	0 (0)	0 (0)	0 (0)
	Silky pocket mouse	*Perognathus flavus*	1	1	1	0 (0)	0 (0)	0 (0)
Leporidae	Desert cottontail	*Sylvilagus audubonii*	10	10	5	5 (50)	7 (70)	3 (30)
Muridae	House mouse	*Mus musculus*	1	1	1	0 (0)	0 (0)	0 (0)
Sciuridae	Rock squirrel	*Otospermophilus variegatus*	27	27	18	7 (25.9)	10 (37)	4 (14.8)
	Spotted ground squirrel	*Xerospermophilus spilosoma*	6	6	5	0 (0)	0 (0)	0 (0)
**Total**			**473**	**458**	**280**	**115 (25.1)**	**304 (66.4)**	**83 (18.1)**

The number of tagged individuals is the number of individuals of a species identified with a unique ID. Test results for the presence of *Trypanosoma* or *Bartonella* DNA are shown as the number of positive samples with the percent prevalence out of the total tested per species in parentheses.

### Patterns of *Trypanosoma* DNA prevalence

**Fig 1:**
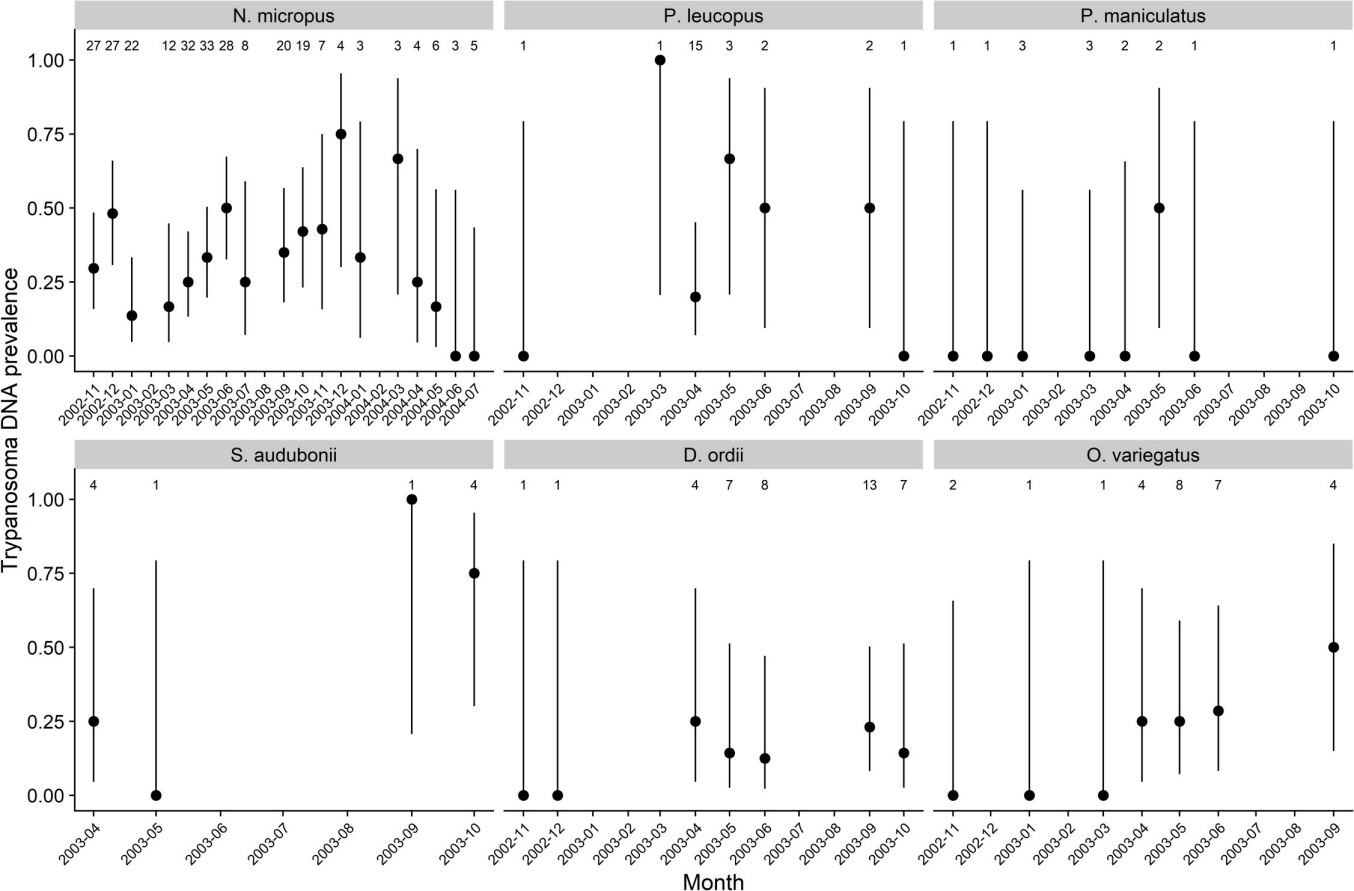
*Trypanosoma* DNA prevalence in positive host species over time. Circles show estimated prevalence and lines show 95% Wilson score confidence intervals. Numbers above each panel show the total individuals of the species tested in a month.

*Trypanosoma* DNA was detected in 115 (25.1%) of the 458 samples tested over the whole study period, including samples from individuals recaptured in multiple months (Table 1). *Trypanosoma* DNA prevalence was lower than *Bartonella* DNA prevalence (66.4%) across all species and dates. Positive species included *N*. *micropus*, *Peromyscus leucopus*, *P*. *maniculatus*, *D*. *ordii*, *Syvilagus audubonii*, and *O*. *variegatus* with *Trypanosoma* DNA prevalence ranging from 7.1–50% (Table 1). Considering all sampled species, there was a significant difference in *Trypanosoma* DNA prevalence among species over the whole study period (χ^2^ = 42.8, df = 13, p-value < 0.001). Within positive species, detection of *Trypanosoma* DNA was not consistent over time due to variation in the number of samples collected in a month (Fig 1). Detection was more consistent for *N*. *micropus* because of the larger number of samples collected from this species across each month, although there was more variation after October 2003 when fewer samples were collected (Fig 1).

**Fig 2:**
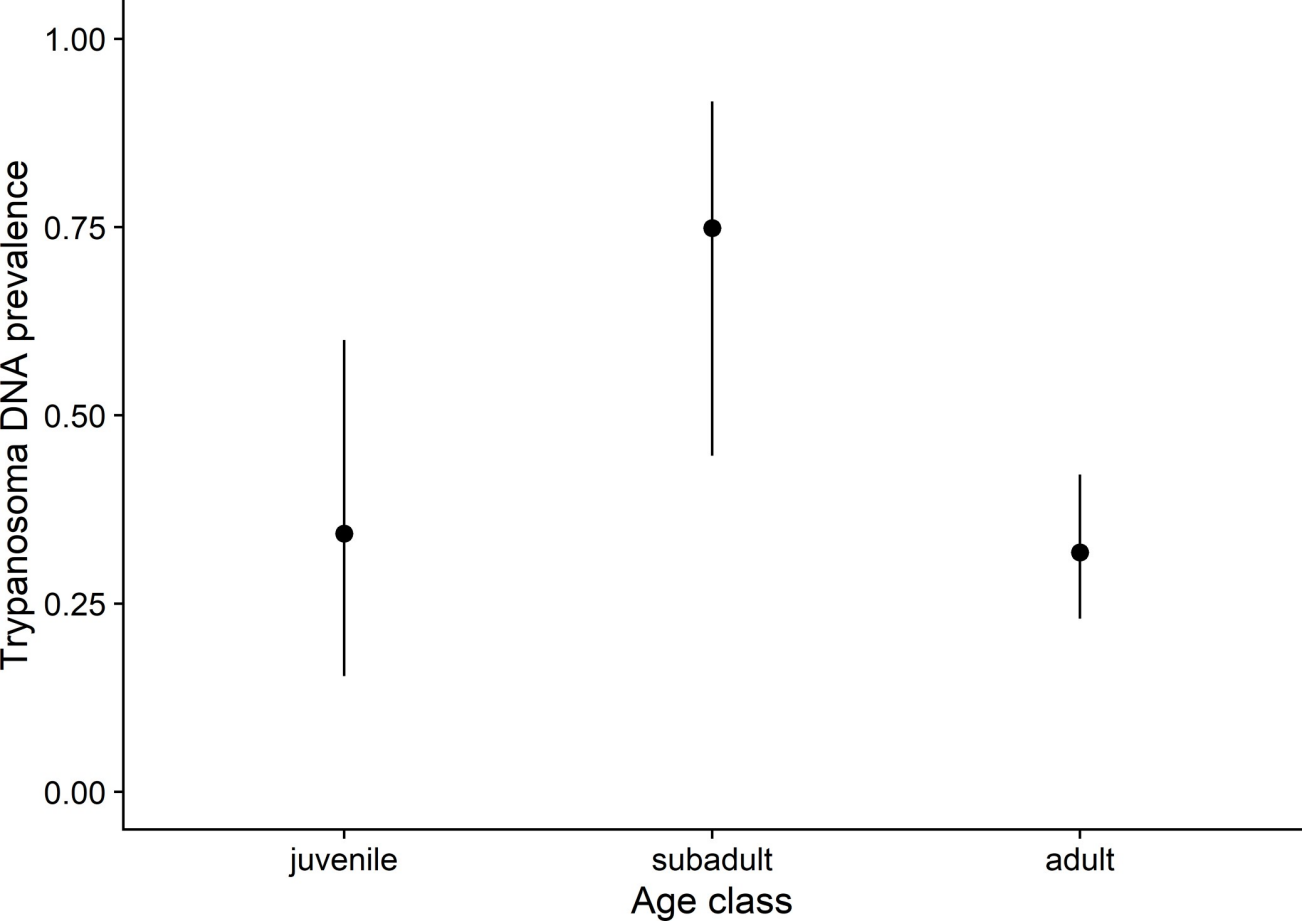
Relationship between *Trypanosoma* infection and age class in *N*. *micropus*. Circles show the estimated prevalence and lines show 95% binomial confidence intervals based on the sample size of tested individuals from each age class. Values are means calculated after 1000 iterations of stratified sampling by individual tag number and model fitting.

Given the large number of samples collected and tested from *N*. *micropus*, we examined available covariates that might explain variation in the probability of *Trypanosoma* infection across captured individuals over the whole study period. We used a combination of stratified sampling by individual tag number and model selection by AICc to determine the best fitting combination of covariates in the model. The model with the lowest mean AICc after 1000 iterations of stratified sampling and model selection contained only age class as a covariate (S1 Table), indicating that subadults were more likely to be infected than juveniles or adults (Fig 2; S2 Table). However, the amount of variation in the probability of *Trypanosoma* infection among *N*. *micropus* explained by the model with age class was low, with a mean R^2^ over the 1000 iterations of only 7.4%. The predictive power of the model was also low, with a mean AUC of 0.592, meaning that the ability of age class to predict the presence of *Trypanosoma* DNA was only marginally better than random.

The effect of higher prevalence in subadults was also observed to a small degree in the variation in prevalence in the whole *N*. *micropus* population over the study period. In three months when higher prevalence was observed (December 2002, June 2003, and September 2003; Fig 1), more than 14% of the captured individuals were subadults. However, the correlation between the prevalence and proportion of subadults across all months was positive but not statistically significant (Pearson’s R = 0.19, p-value = 0.44).

### *Trypanosoma*-*Bartonella* coinfection

Considering all host species together, *Bartonella* and *Trypanosoma* infections were not correlated. The observed coinfection prevalence was 18.1%, which is not statistically different (χ^2^ = 0.7, df = 1, p-value = 0.4) from the expected 16.7% based on the prevalence of *Bartonella* DNA (66.4%) and *Trypanosoma* DNA (25.1%). For each of the host species with more than one individual infected with *Trypanosoma* over the whole study period, we saw no significant association based on binomial tests of proportions (p-value > 0.05) between *Bartonella* and *Trypanosoma* infection (S3 Table). We also saw no significant association between infections in species across months of the study period when more than one individual was captured and tested positive for either organism (S4 Table).

### *Trypanosoma* species diversity

**Fig 3:**
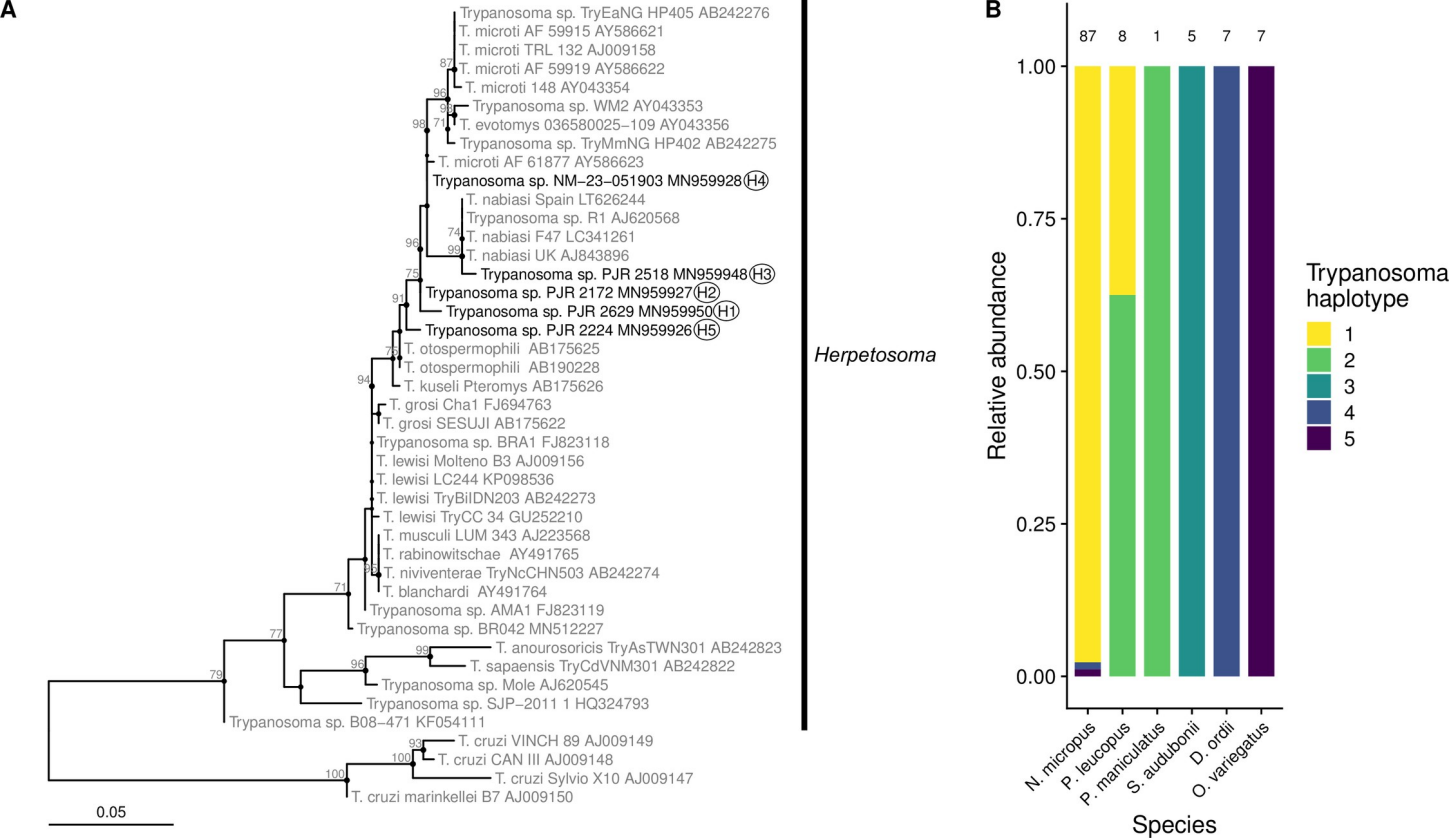
Phylogenetic tree and host associations of *Trypanosoma* haplotypes. (A) The maximum likelihood tree was produced from a 559 bp alignment (including gaps) of the 18S rRNA gene using the top model (TIM2+F+R2) following model selection with AICc in IQ-Tree. Reference sequences are colored gray and new haplotypes are colored black. Branch support values estimated from 1000 ultrafast replicates are shown next to branches if they were greater than 70%. Sequences representing *T*. *cruzi* were included as an outgroup to the other sequences in the *Herpetosoma* subgenus. Branch lengths are in units of the number of base substitutions per site. (B) The relative abundance of haplotypes identified in sampled animals are summarized for each positive host species. Numbers above the panel show the total *Trypanosoma* sequences obtained for each species.

Phylogenetic analysis of positive 18S rRNA sequences recovered five *Trypanosoma* haplotypes that were distinct from one another and from reference *Trypanosoma* species on GenBank (Fig 3). All identified haplotypes clustered within the *Herpetosoma* subgenus (*T*. *lewisi* clade) along with species *T*. *nabiasi*, *T*. *microti*, *T*. *evotomys*, *T*. *otospermophili*, and *T*. *kuseli*. Haplotype 1 sequences were found in two species in the family Cricetidae, *N*. *micropus* (85) and *P*. *leucopus* (3). Haplotype 1 was most closely related to haplotype 2 with 98.5% sequence identity (450/457 bp) although their phylogenetic relationship was not resolved. Haplotype 2 was found in two *Peromyscus* species, *P*. *leucopus* (5) and *P*. *maniculatus* (1). Haplotype 3 was strictly associated with *S*. *audubonii* and was most closely related to *T*. *nabiasi* previously detected in European rabbits (*Oryctolagus cuniculus*) and associated ectoparasites in Spain and the UK with 99.6% sequence identity (451/453 bp) and 99% bootstrap support (Fig 3). Haplotype 4 was found predominantly in *D*. *ordii* (7) with one sequence from *N*. *micropus*. This haplotype shared 99.8% sequence identity (452/453 bp) with haplotype 2 and *T*. *microti* detected in *Microtus miurus* in Alaska, USA (GenBank accession AY586623) and 99.6% sequence identity (452/454 bp) with *T*. *microtus*, *T*. *evotomys*, and other *Trypanosoma* haplotypes detected in voles and lemmings (*Myodes*, *Microtus*, and *Lemmus* spp.) from Japan, the UK, and Alaska, USA (AB242276, AB242275, AY043356, AY586622, AY586621, AJ009158, AJ009158). However, the phylogenetic relationship between haplotype 4 and its close relatives is uncertain, demonstrated by the polytomy in this region of the tree (Fig 3). Haplotype 5 was predominantly associated with *O*. *variegatus* (7) with one sequence from *N*. *micropus*. This haplotype shared 99.3% sequence identity (453/456 bp) with *T*. *otospermophili* detected in ground squirrels (*Spermophilus* and *Urocitellus* spp.) imported into Japan from the USA (AB175625, AB190228) and 98.7% sequence identity (450/456 bp) with *T*. *kuseli* detected in the flying squirrel *Pteromys volans* imported into Japan from China (AB175626). However, haplotype 5 is a distinct lineage from *T*. *otospermophili* and *T*. *kuseli* in the phylogenetic tree (Fig 3). The sequences of haplotype 4 and 5 from *N*. *micropus* were detected from animals captured in May 2003 and the sequences of haplotype 1 from *P*. *leucopus* were amplified from animals from April 2003 (S1 Dataset). No sequences of *T*. *cruzi* were amplified with the nested 18S rRNA primers and none of the samples were positive for the qPCR assay specific to *T*. *cruzi* minicircle DNA. Representative sequences amplified from each host species and used to define new haplotypes were uploaded to GenBank (S5 Table). Information on *Trypanosoma* species used as references in the phylogenetic analysis can be found in S6 Table. The trimmed sequences from all new haplotypes submitted to GenBank and *Trypanosoma* references and used for phylogenetic analysis are included in S5 and S6 Tables.

### *Trypanosoma* in recaptured *Neotoma* woodrats

**Fig 4:**
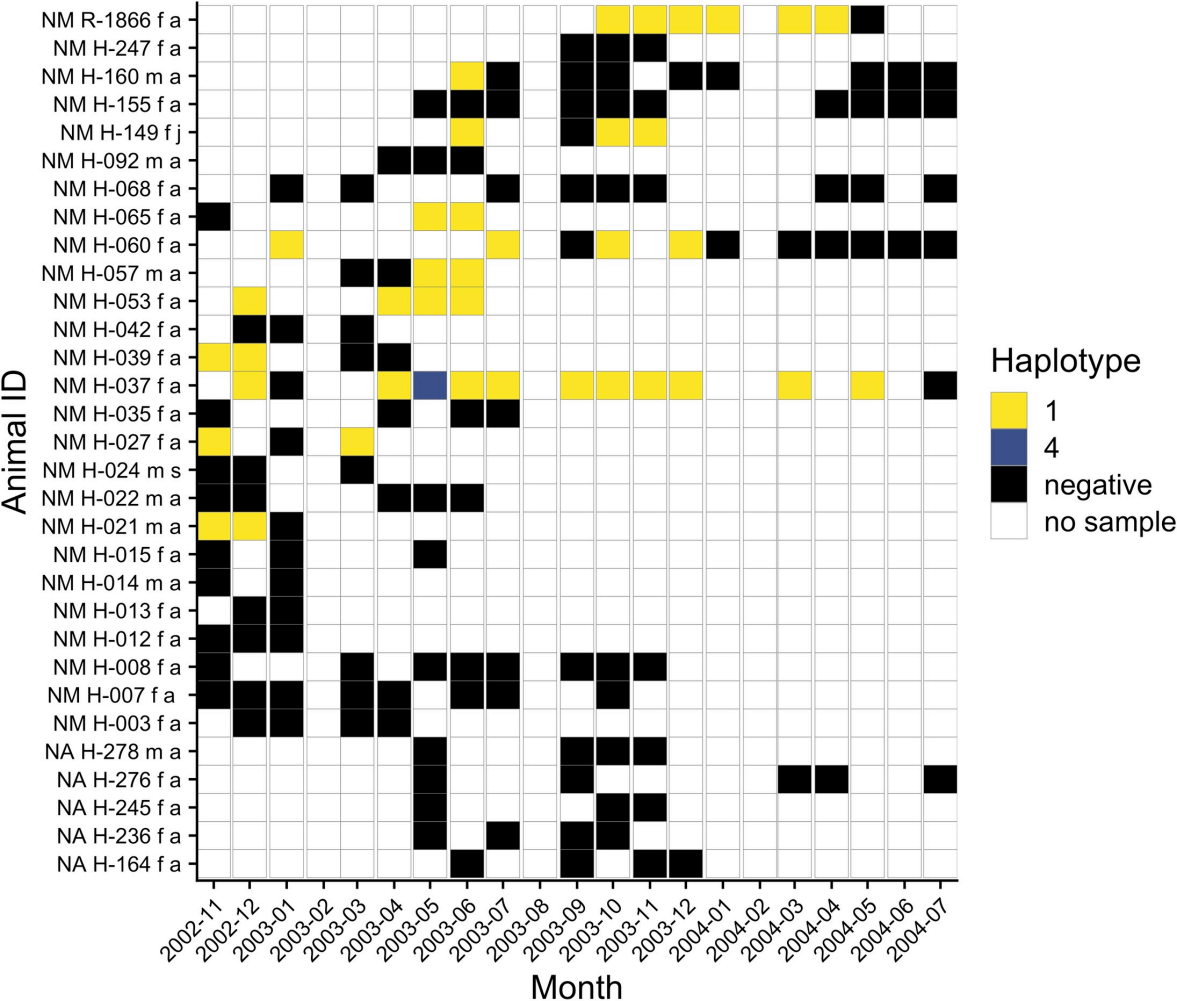
Resampling history and *Trypanosoma* haplotypes in 31 woodrats (NM = *Neotoma micropus* and NA = *Neotoma albigula*) captured three and more times during the 21 sampling months of the study. The *Trypanosoma* haplotypes amplified from infected woodrats in a sample month are shown with different colors.

A total of 31 *Neotoma* woodrats (26 *N*. *micropus*, 5 *N*. *albigula*) were recaptured three or more times over the course of the study. Recaptured animals showed a sparse pattern of *Trypanosoma* infection, with 20 individuals showing no infection over the study period and the remaining 11 showing a repeating pattern of infection with the same *Trypanosoma* haplotype over multiple months of sampling (Fig 4). Positive *N*. *micropus* individuals were predominantly infected with haplotype 1, occasionally interrupted by a negative test or, in one case (adult female *N*. *micropus* H-037), detection of haplotype 4 in May 2003.

## Discussion

The aim of the present study was to investigate the prevalence and genetic diversity of *Trypanosoma* parasites in a community of rodents and lagomorphs in New Mexico, document patterns of coinfection in hosts with trypanosomes and *Bartonella* bacteria, and determine factors that predict infection risk in an abundant species in the community, *N*. *micropus*. We found that that the estimated prevalence of *Trypanosoma* DNA in species ranged from 7.1% in *P*. *leucopus* to 50% in *S*. *audubonii*, with consistent prevalence around 30% for *N*. *micropus* throughout the study period. All *Trypanosoma* haplotypes sequenced in this study clustered in the *Herpetosoma* subgenus (*T*. *lewisi* clade) along with *T*. *kuseli*, *T*. *evotomys*, *T*. *otospermophili*, *T*. *microti*, and *T*. *nabiasi* and were largely specific to one host or two phylogenetically related hosts (Fig 3). We also found that the probability of *Trypanosoma* infection in *N*. *micropus* varied by age class, with a higher prevalence in subadults compared to juveniles and adults. We provide evidence that *N*. *micropus* individuals can be persistently infected over multiple months with the same *Trypanosoma* haplotype. Finally, coinfection patterns with *Bartonella* bacteria did not indicate that *Trypanosoma* infections are facilitated by infection with *Bartonella* in *N*. *micropus* or across sampled host species.

The phylogenetic position of the *Trypanosoma* haplotypes enables prediction of their natural transmission cycle [69]. Trypanosomes in the *T*. *lewisi* clade are believed to be transmitted by fleas, with supporting experimental evidence for *T*. *grosi*, *T*. *lewisi*, *T*. *microti*, *T*. *nabiasi*, and *T*. *evotomys* [12, 69, 70]. Animals contract infection through wounds contaminated with infected flea feces or by ingesting infected fleas and flea feces during grooming [1, 71]. Fleas become infected upon ingesting a blood meal from an infected animal [71]. As all obtained sequences cluster within the *T*. *lewisi* clade, their potential vectors could be among 107 local flea species [72]. However, experimental manipulation of flea prevalence demonstrated vector-independent transmission of *T*. *microti* in field voles (*Microtus agrestis*) [73], suggesting that direct transmission between hosts may also be important.

Due to the high variation in *Trypanosoma* DNA prevalence among host species and the observed pattern of host-specificity, comparing prevalence rates is more feasible at the host species or genus level, rather than in the whole community of rodents and lagomorphs. We discuss our results in the context of previous surveys of trypanosomes in each species or related species in the United States.

### *Trypanosoma* spp. in *Neotoma* woodrats

The observed *Trypanosoma* prevalence in our sampled population of *N*. *micropus* is comparable to estimates in *Neotoma* woodrats in other areas. The only prior study in New Mexico found one *N*. *micropus* and one *N*. *albigula* positive for *T*. *cruzi* [23]. However, it is unclear whether the methods used in their study (xenodiagnosis in triatomine bugs) could have detected other trypanosomes besides *T*. *cruzi*. In California, *T*. *neotomae* was described from 19.4% of *N*. *fuscipes* [29] and later found in *N*. *lepida* there as well [74]. In Texas, *T*. *cruzi* prevalence in *N*. *micropus* ranged from 21% to 46% [22, 24–26, 75]. Studies in Louisiana and California revealed *T*. *cruzi* prevalence of 73.3% in *N*. *floridana* [27] and 14.3% in *N*. *macrotis* [28]. Again, these studies used laboratory methods that are specific for *T*. *cruzi* detection, so it is unknown whether other *Trypanosoma* species were truly absent in the tested woodrats. Studies in Texas also identified *T*. *neotomae*, *T*. *neotomae*-like, and *T*. *lewisi*-like parasites in *N*. *micropus* [1, 22, 76]. A recent molecular study in Texas found that 24% of *N*. *micropus* were infected with *T*. *neotomae*-like parasites [76]. Comparison of partial sequences of the 24S*α* gene showed that the *T*. *neotomae*-like trypanosomes shared 99.1% sequence identity compared with *T*. *kuseli* and *T*. *otospermophili* [76]. In Kansas, 13% of *N*. *floridana* carried *T*. *kansasensis* [30]. An interesting outcome of our study was that all 47 tested *N*. *albigula* samples were negative for *Trypanosoma* species. However, *T*. *cruzi* was reported in *N*. *albigula* in Arizona and New Mexico and *T*. *neotomae* was detected in Mexico [23, 77].

Considering that we used specific PCR primers that targeted *T*. *cruzi* and more general primers for detection of other *Trypanosoma* species, we believe that our results indicate that *T*. *cruzi* is likely absent or at very low prevalence in our studied rodent community, and that *Trypanosoma* sp. haplotype 1, belonging to the *T*. *lewisi* clade, is prevalent in *N*. *micropus*. Since there have been no other studies that have targeted the 18S rRNA gene in *Neotoma* woodrats, it is challenging to interpret the identity of *Trypanosoma* haplotype 1 in relation to other *Trypanosoma* species identified in *Neotoma* spp. While the prevalence values estimated in our study compare favorably with estimates of *T*. *neotomae* or *T*. *neotomae*-like parasites in California and Texas [29, 76], we cannot determine whether the haplotype found in *N*. *micropus* in New Mexico is *T*. *neotomae* or another species without morphological examination of the trypanosomes. Such examination was not possible with the archived samples in our study, so additional studies using both morphological examination of trypanosomes in fresh blood smears and molecular sequencing will be necessary to conclusively identify these parasites in *N*. *micropus*. Furthermore, broader surveys using such methods in other *Neotoma* species may recover *T*. *kansasensis* or other *Trypanosoma* species occurring in the US.

### *Trypanosoma* spp. in other rodents

This study also provided evidence of *Trypanosoma* infection in several other rodent species for the first time, including *D*. *ordii* and *O*. *variegatus* [23, 76]. Previous studies have detected *T*. *otospermophili*, *T*. *cruzi*, and a *T*. *lewisi*-like species in *O*. *beecheyi* [78–82], a congener of *O*. *variegatus*. *Trypanosoma* haplotype 5 found in *O*. *variegatus* in our study is genetically similar to *T*. *otospermophili* and *T*. *kuseli*, both species associated with sciurid rodents, although additional sequencing and morphological examination will be necessary to ascertain whether haplotype 5 represents a new *Trypanosoma* species.

For other species that were negative for *Trypanosoma* DNA in our study, the limited number of samples from these species prevents us from adequately assessing their host status. More studies with increased sampling from *O*. *leucogaster*, *P*. *truei*, *P*. *flavus*, *R*. *megalotis*, *D*. *spectabilis*, *M*. *musculus*, and *X*. *spilosoma* will be necessary to determine if trypanosomes are truly absent from these species in New Mexico. *T*. *cruzi* has been reported from *M*. *musculus* [22, 76] and *O*. *leucogaster* [25] in Texas, as well as *P*. *truei* and *R*. *megalotis* in California [83, 84]. The single *P*. *leucopus* previously tested from Texas was negative for *T*. *cruzi* and other trypanosomes [76]. *P*. *maniculatus* was *T*. *cruzi*-negative in New Mexico and California [23, 78], but *T*. *peromysci* was found in this host in Canada [85] and Arizona [77]. Like the relationship of *Trypanosoma* haplotype 1 from *N*. *micropus* and *T*. *neotomae* or *T*. *kansasensis*, the question of whether haplotype 2 found in *P*. *leucopus* and *P*. *maniculatus* is *T*. *peromysci* will require additional sampling, morphological characterization of trypanosomes, and molecular sequencing.

### *Trypanosoma* spp. in lagomorphs

Our study found that *S*. *audubonii* carried *Trypanosoma* haplotype 3, which was closely related in *T*. *nabiasi*. First discovered in the blood of European rabbits (*Oryctolagus cuniculus*) in France [86], *T*. *nabiasi* has been reported in wild *O*. *cuniculus* in Europe [34], domesticated *O*. *cuniculus* outside Europe [1], and introduced *O*. *cuniculus* in Australia [33]. In Spain, *T*. *nabiasi* prevalence in *O*. *cuniculus* was reported as high as 82.4% [32, 87]. Trypanosomes morphologically similar to *T*. *nabiasi* have previously been detected in North American cottontails (*Sylvilagus audubonii*, *S*. *nuttallii*, *S*. *floridanus*) [85, 88, 89], but were unnamed and have not previously been sequenced. Despite the introduction of *O*. *cuniculus* to the USA and its current widespread status [90], the sequence distance between haplotype 3 and *T*. *nabiasi* (99.6%) suggests that these organisms may be distinct species. Sequences from trypanosomes in introduced *O*. *cuniculus* in Australia were identical to *T*. *nabiasi* in Europe, likely the result of a recent introduction of the parasites in rabbit fleas or infected rabbits [33]. Such a recent introduction does not appear to explain the origin of *Trypanosoma* haplotype 3. Instead, a more parsimonious explanation is that *T*. *nabiasi* and haplotype 3 diverged along with their hosts when the genera *Oryctolagus* and *Sylvilagus* separated between 7 and 16 million years ago [91–93]. More studies on trypanosomes in native and introduced lagomorphs in the United States will help to clarify the evolutionary history and host associations of *T*. *nabiasi* and related species.

Another argument against a recent introduction of *T*. *nabiasi* is that the rabbit flea *Spilopsyllus cuniculi*, the vector of *T*. *nabiasi* in Europe [33], does not occur in the United States [94]. However, a congener, *S*. *inaequalis*, was collected from wild lagomorphs in New Mexico [72]. In our study community, *S*. *audubonii* harbored the flea species *Euhoplopsyllus glacialis*, *Cediopsylla inaequalis*, anrd *Orchopeas sexdentatus*. *E*. *glacialis* fleas parasitize cottontails but can also invade buildings and bite humans [95]. Therefore, given the potential for atypical human infections by trypanosomes in the *T*. *lewisi* clade, more studies could determine the vectors of trypanosomes in *S*. *audubonii* and evaluate risks for human exposure.

### *Trypanosoma* infection dynamics in *Neotoma micropus*

Our focused analysis on the large number of samples collected from *N*. *micropus* found that *Trypanosoma* prevalence varied with age class, with a higher prevalence in subadults than juveniles or adults. This pattern of infection may partially explain some months of higher prevalence in the *N*. *micropus* population when the proportion of subadults among the captured animals was higher, but this trend was weak over the whole study period. The relationship between age class and prevalence over time was likely weakened by variation in sampling intensity (especially after October 2003) and whether an animal with a persistent infection was captured in a given month.

Previous studies have observed relationships between *Trypanosoma* prevalence and rodent age or weight, although the shape of the relationship is not certain. *T*. *musculi*-like infection was found in immature rather than adult rodents [64] and *T*. *evotomys* infection was detected predominantly among older rodents [65]. Other authors found age played only a minor role [66, 67]. The most similar results to our study were from *T*. *microti* in voles in the UK, showing a convex relationship where prevalence first increases with age/weight then later decreases [19]. A relationship of this shape suggests that susceptibility or exposure to trypanosomes varies over an animal’s lifetime and that animals may develop some level of immune protection from reinfection later in life.

Evidence of a protective effect of past trypanosome infection has been documented for multiple *Trypanosoma* species in rodents. Specifically, the presence of persistent dividing forms of *T*. *musculi* in the kidneys of previously infected *M*. *musculus* was associated with high antibody titers and a lack of parasitemia following a second challenge with *T*. *musculi* [96]. These persisting forms of *T*. *musculi* in the kidney could be observed almost a year after recovery from parasitemia [97, 98]. Persistent dividing forms of *T*. *grosi* in the kidneys of Mongolian jirds (*Meriones unguiculatus*) following the clearance of trypanosomes from the blood appear to have similar protective role. However, pregnancy may lead to reactivation of *T*. *musculi* persisting in the liver of *M*. *musculus*, producing short bouts of parasitemia [99].

Our data suggest *Trypanosoma* infections in *N*. *micropus* appear to be persistent in some individuals. Ten of the 26 *N*. *micropus* recaptured more than three times in our study were positive for the same *Trypanosoma* haplotype over multiple months (Fig 4). Unfortunately, due to our exclusive use of molecular detection of trypanosome DNA in blood, we cannot determine whether the persistent infections we observed are patent parasitemia or simply detection of DNA from trypanosomes persisting in the kidneys or another organ in these individuals.

For the one individual (H-037) that was positive for haplotype 4 in May 2003, it is possible that this animal was briefly infected with haplotype 4 circulating in *D*. *ordii* in this community, possibly via contact between *N*. *micropus* and *D*. *ordii* or sharing of ectoparasites. The animal may have been coinfected with haplotype 1 at this time, but only haplotype 4 was detected. Repeated PCR and sequencing from this sample confirmed detection of haplotype 4. A previous study of multiple rodent species showed that animals that were serially challenged with two different *Trypanosoma* species had lower levels of parasitemia and shorter patent periods than control animals challenged only once [100]. A cross-protective immune response activated by an initial infection might explain the short duration of haplotype 4 infection in individual H-037. However, we could not confirm the coinfection with a sequence of haplotype 1 and cannot rule out the possibility of sample contamination during DNA extraction.

Despite these uncertainties, our findings provide some important information on the dynamics of *Trypanosoma* infection in natural populations of *N*. *micropus*. The decline in prevalence among adults might be partly explained by some immune protection from reinfection after recovery from parasitemia earlier in life. Persistence of trypanosomes in the liver or other organs could be involved in the stimulation of a protective antibody response but may result in occasional relapse of parasitemia during pregnancy or other forms of stress. Obviously, additional longitudinal studies that monitor individuals for a much longer period would be able to track how the force of infection varies with age with more accuracy than the age classes we have used. Additionally, analysis of parasitemia and persistent infection in organs would require collection of fresh blood and tissue. Measures of antibody levels in previously infected individuals, especially during reproduction or periods of nutritional stress, might be able to explain the reactivation of persistent infections or reinfection in adult *N*. *micropus*.

### Patterns of *Trypanosoma*-*Bartonella* coinfection

Our findings of coinfection by *Bartonella* and *Trypanosoma* in multiple species are in line with other studies of these pathogens in rodents. Prior studies detected *Bartonella* coinfections with *T*. *evotomys*, *T*. *microti*, and *T*. *grosi* in voles from Norway [101] and with *T*. *musculi*-like species in 26.7% of British rodents [64]. Studies in Poland revealed *Trypanosoma*-*Bartonella* coinfection rates from 3.1% to 17.3% in voles [66, 67, 102]. The lack of association between *Trypanosoma* and *Bartonella* infection may indicate that these two pathogens do not compete for host resources or facilitate infection through immune suppression, but additional work using experimental infections would be useful to understand how these parasites interact with one another and the host’s immune system [103].

## Limitations

One important limitation of our study is that we relied solely on molecular detection of *Trypanosoma* DNA to assess prevalence in host species over time, thus we could not determine whether a positive individual had living trypanosomes in their blood at the time of sampling. Living trypanosomes could have been observed in fresh blood smears prepared at the time of collection, but this was not done in our study and would not have been possible from the archived samples. As already mentioned, these smears would also have been useful in describing *Trypanosoma* haplotypes using morphological features, thereby allowing us to reconcile the observed haplotypes with *Trypanosoma* species previously described in some of our study species but not sequenced.

Another drawback of our methodology was the lack of internal controls to determine whether DNA extraction from blood was successful. Since the main focus of our study was estimation of *Trypanosoma* prevalence and diversity, the potential presence of false negatives was not important. However, internal controls would have been useful in the analysis of infections in recaptured *Neotoma* woodrats. For example, four of the ten positive recaptured *N*. *micropus* (H-027, H-037, H-060, H-149; Fig 4) showed a pattern of positive-negative-positive over multiple months. The negative tests might have been due to the absence of trypanosomes from blood (or at a level below the detection limit of PCR) or due to a failure in DNA extraction. An internal control PCR targeting host DNA present in blood might have been able to distinguish between the different causes of a negative test for *Trypanosoma* DNA. With the inclusion of internal controls and more consistent recapture of animals over a longer period of time, future longitudinal studies of *Trypanosoma* infection in natural host populations would be able to measure the duration of infection in individual animals.

Our study also used only one marker for DNA sequencing. While sequences of the 18S rRNA gene have frequently been used for the detection and identification of *Trypanosoma* species, the information contained in these short sequences is insufficient to reconstruct deeper evolutionary relationships among *Trypanosoma* species. Sequencing of additional markers (e.g., 70 kDA heat-shock protein, gGADPH) from the haplotypes identified in our study and from other species in the *T*. *lewisi* clade would help to clarify the evolutionary history of this group [7, 104]. Deep sequencing methods could also be beneficial for detecting coinfections of different *Trypanosoma* haplotypes [4, 105].

Finally, we recognize that differentiation between some rodent species based on morphological observations in the field is known to be challenging in this study area, particularly within the genera *Neotoma* and *Peromyscus*. We removed some *Peromyscus* mice from the study because they could not be identified to the species level, but it is possible that some other *Neotoma* woodrats or *Peromyscus* mice were misidentified. While these errors would have had minimal effect on our conclusions about *Trypanosoma* associations at the host family or genus level, uncovering more subtle patterns of host specificity among trypanosomes would require more robust methods of host identification. We recommend that future studies in this area use molecular methods (e.g., barcoding) to confirm identification of host species. These data, along with sequencing of additional markers from trypanosomes, may reveal additional host specificity between *Trypanosoma* haplotypes and host species, subspecies, or regional populations.

## Conclusions

Rodents and lagomorphs of New Mexico host a variety of host-specific *Trypanosoma* haplotypes. *Trypanosoma* infection in *N*. *micropus* had consistent prevalence around 30% in the population over time, with varying prevalence across age groups, and some *N*. *micropus* appeared to have persistent infections with the same haplotype over multiple months. We report the first molecular detection of a *Trypanosoma* haplotype related to *T*. *nabiasi* in North America in the blood of *S*. *audubonii* and the first detection of *Trypanosoma* infection in *D*. *ordii* and *O*. *variegatus*. While the pathogenicity of the trypanosomes reported in this study is unknown in humans, the *Trypanosoma* species present in this rodent and lagomorph species should be monitored as potential agents of human trypanosomiasis.

## Supporting information

S1 Table Generalized linear models ranked by AICc following 1000 iterations of stratified sampling and model selection. “FleaPos” represents the presence/absence fleas at the time of capture while “Fleas” is the number of fleas at capture. “Bartonella” is the presence of *Bartonella* DNA and “Sex*Weight” is an interaction term for *N*. *micropus* sex and weight.
(XLSX)

S2 Table Model coefficients and 95% confidence intervals for top GLM from S1 Table containing age class as a main effect. (XLSX)

S3 Table Observed and expected *Bartonella-Trypanosoma* coinfection rates in captured rodent and lagomorph species across all sampling time points. (XLSX)

S4 Table Observed and expected *Bartonella-Trypanosoma* coinfection rates in captured rodent and lagomorph species across months when more than one individual was sampled and tested positive for either infection. (XLSX)

S5 Table GenBank accession numbers and host information on sequences representing new *Trypanosoma* haplotypes identified in captured rodents and lagomorph species from this study. (XLSX)

S6 Table GenBank accession numbers and host information on reference *Trypanosoma* species used for phylogenetic analysis. (XLSX)

S1 Dataset Geographic coordinates, covariate information, *Trypanosoma-Bartonella* testing results, and identified *Trypanosoma* haplotype for all captured animals. (XLSX)
